# Event-related potentials and behavioral correlates of emotional recognition memory in late pregnancy

**DOI:** 10.1007/s00737-024-01503-8

**Published:** 2024-08-21

**Authors:** Sivan Raz

**Affiliations:** 1 Department of Psychology, The Per Sternberg EEG-ERP Laboratory for the Study of Brain and Behavior, Tel Hai College, Upper Galilee, Israel; 2 Department of Behavioral Sciences, The Center for Psychobiological Research, The Max Stern Yezreel Valley College, 19300 Emek Yezreel, Israel

**Keywords:** Late pregnancy, Recognition memory, Emotional words, Event-related potentials

## Abstract

**Purpose:**

Research on cognitive and emotional functions during pregnancy challenges the prevalent perception of cognitive decline in pregnant women. This study investigates the behavioral and neural dynamics of cognitive-affective processing in third-trimester pregnant women, comparing them with non-pregnant controls.

**Methods:**

Using a 64-channel EEG-ERP system, we recorded brain activity as participants engaged in an emotional word recognition task. This task involved initially viewing a sequence of emotional and neutral words, followed by a recognition test where participants identified each word as 'new' or 'previously seen'.

**Results:**

Contrary to widespread beliefs about diminished recognition ability during late pregnancy, our results revealed no significant differences in error rates between groups. However, pregnant participants demonstrated slower reaction times. In terms of neural responses, pregnant women exhibited increased amplitudes in the N1, P2, and N400 ERP components, suggesting that they may require additional brain resources compared with non-pregnant individuals to process perceptual information. A significant interaction was observed between pregnancy status and the emotional valence of stimuli. Pregnant women showed heightened N1 and N400 responses to negative words, indicating increased sensitivity to stimuli potentially representing threat. This enhanced response was not observed for positive or neutral words. Furthermore, there was an amplified N1 response to 'new' words, but not to 'old' words.

**Conclusion:**

These findings suggest that late pregnancy is characterized by heightened responsiveness to new and particularly negative stimuli, potentially leading to a more cautious behavioral approach. Heightened vigilance and sensitivity could offer evolutionary advantages, optimizing fetal development and enhancing maternal well-being.

## Introduction

A growing body of research supports the notion of cognitive-affective changes during pregnancy. Studies where pregnant women self-assess cognitive abilities (attention, concentration, memory) indicate a perceived decline during pregnancy and postpartum (Brett and Baxendale 2001; Crawley et al. 2003, 2008; Christensen et al. 1999; Henry and Rendell 2007; Janes et al. 1999). However, empirical research only partially supports these perceptions (for reviews see: Davies et al. 2018; Brown and Schaffir 2019; Ouellette and Hampson 2019). Cognitive decline during pregnancy has been attributed to factors like mood changes, anxiety, sleep quality alterations, energy trade-offs, and hormonal fluctuations (Ziomkiewicz et al. 2019). Evidence indicates the pregnancy trimester as a significant factor, although findings vary. Some research points to more marked cognitive deficits in the second trimester (Brindle et al. 1991), while other studies suggest impairments across all trimesters (de Groot et al. 2006; Sharp et al. 1993) or particularly in the third trimester (Davies et al. 2018; Henry and Sherwin 2012; Keenan et al. 1998; Wołyńczyk-Gmaj et al. 2022). A recent meta-analysis (Davies et al. 2018) concluded that cognitive function in pregnant women, especially during the third trimester, is poorer compared to controls. However, the effect sizes were small to moderate, with both groups performing within normal memory and cognitive function ranges.

Memory function during pregnancy emerges as a key area of interest. Empirical studies focusing on memory function during pregnancy have yielded inconsistent results. Pregnant women often report a decline in memory through self-report questionnaires, a finding supported by various studies (Brett and Baxendale 2001; Buckwalter et al. 1999; Crawley et al. 2008; De Groot et al. 2006; Farrar et al. 2014; Keenan et al. 1998; Macbeth and Luine 2010; Rendell and Henry 2008). However, this is not a universal finding; several studies have reported no significant pregnancy-related memory impairment (Casey 2000; Casey et al. 1999; Crawley et al. 2003; Logan et al. 2014; McDowall and Moriarty 2000; Onyper et al. 2010), and some have even observed improved memory performance during pregnancy, indicating potential cognitive advantages (Anderson and Rutherford 2011, 2012; Callaghan et al. 2022; Christensen et al. 1999; Macbeth et al. 2008). Animal studies also suggest links between pregnancy, motherhood, and enhanced spatial learning and memory functions (Galea et al. 2000; Gatewood et al. 2005). A decline in memory during pregnancy is usually observed when using free recall testing methods (Buckwalter et al. 1999; de Groot et al. 2006; Henry and Rendell 2007; Keenan et al. 1998), however, the results are mixed regarding recognition tasks. Some studies found no differences between pregnant women and controls (Henry and Rendell 2007; Sharp et al. 1993), while others suggest that pregnancy may enhance recognition memory (Brindle et al. 1991; Christensen et al. 1999; Mickes et al. 2009). Self-reported memory complaints during pregnancy typically involve declarative memory, a system that includes conscious recall or recognition of facts and events (Cohen and Squire 1980; Graf and Schacter 1985). The present study focuses on emotional recognition memory during late pregnancy, a subcategory of declarative memory. Recognition memory assessment involves categorizing items as 'old' (previously studied) or 'new'. This performance can be based on familiarity or recollection processes, where familiarity is a fast, automatic assessment of memory strength without contextual retrieval, and recollection involves slow, effortful retrieval of qualitative information about a studied event (Atkinson and Juola 1973; Humphreys et al. 1989; Jacoby and Dallas 1981; Mandler 1980; Yonelinas 2002; Yonelinas et al. 2005, 2010; Kipp et al. 2015; Stróżak et al. 2016).

Current understanding of pregnant women's emotion processing style is limited. Empirical research indicates that pregnancy-related sex hormones, such as estrogen and progesterone, which increase from early to late pregnancy, may influence emotion-processing systems and enhance sensitivity to emotional content (Jasnow et al. 2006; Pearson et al. 2009). Pearson et al. (2009) conducted a study involving women in early pregnancy (before the 14th gestational week) and again in late pregnancy (after the 34th gestational week). They employed an emotional recognition task and a clinical interview to assess changes in emotional processing. Their findings suggest that women in late pregnancy exhibit an improved ability to recognize and encode emotional faces. Additionally, they observed that anxiety symptoms were correlated with greater accuracy in encoding faces that convey threat, such as fearful and angry expressions. Specifically, during late pregnancy, women demonstrated heightened accuracy in recognizing expressions indicating threat or harm, including fearful, angry, and disgusted faces, when compared to their abilities in early pregnancy.

Despite significant public and scientific interest in cognitive-affective functions among pregnant women and the associated stigmas, there remains a limited understanding of the neurofunctional and neuroanatomical changes during human pregnancy (for reviews see Cárdenas et al. 2019; Pawluski et al. 2022). Research on pregnancy-related brain changes primarily derives from basic laboratory animal studies (Jasnow et al. 2006; Kinsley et al. 2006; Macbeth and Luine 2010) and some non-invasive human imaging studies (Dinc et al. 1998; Oatridge et al. 2002; Roos et al. 2011).

Event-Related Potentials (ERPs) present an ideal, non-invasive, and safe method for studying pregnancy, providing exceptional temporal precision and resolution in tracking brain dynamics associated with sensory, cognitive, and emotional processing. Despite these advantages, the utilization of ERPs in pregnancy research remains limited (Ali et al. 2018; Begum and Reza 2021; Fiterman and Raz 2019; Olofsson et al. 2005; Raz 2014; Rutherford et al. 2016, 2017, 2018, 2019, 2021; Tandon et al. 1996). Importantly, most of these ERP studies did not explore cognitive functioning. For example, the studies conducted by Rutherford et al. (2016, 2017, 2018, 2019, 2021) examined ERPs in response to infant faces or cries, and Olofsson et al. (2005) examined olfactory and chemosomatosensory function. Among the few studies that did focus on cognitive function, only two specifically investigated cognition-related ERPs elicited by visual stimuli (Fiterman and Raz 2019; Raz 2014). These investigations evaluated sustained attention and inhibitory control during late pregnancy, utilizing visual oddball and stop-signal tasks. The findings revealed cognitive changes during pregnancy, accompanied by modifications of several ERP components' amplitudes, such as P1, N170, and P300. These findings underscore the need for more extensive research to explore the neural foundations underlying cognitive and emotional functions during pregnancy. To date, there are no studies examining ERPs during a memory recognition task in pregnant women.

The current study aimed to investigate the behavioral (accuracy, reaction time; RT) and neural (ERPs) correlates of cognitive-affective processing in late pregnancy. Specifically, we sought to answer two questions at both behavioral and neural levels: (1) Do pregnant women, compared to matched non-pregnant controls, react differently to emotional (negative/positive) and neutral words? (2) Do pregnant women, compared to a control group, respond differently to 'old' (previously presented) versus 'new' words? Cognitive function (recognition memory) and neural activity were evaluated using scalp-recorded ERPs during an emotional word recognition task. This task involved an initial presentation of a continuous sequence of emotional and neutral words, followed by a recognition memory test where participants had to indicate for each word whether it was 'new' or 'old'. To our knowledge, this is the first study to examine both behavioral and neural indices of recognition memory and related processing of verbal emotional content in pregnant women using ERPs.

The most intensively studied language-related ERP component is the N400. It is a negative-going voltage deflection (tends to be largest over centro-posterior sites) starting around 250 ms and peaking around 400 ms after the onset presentation of a word, sentence, or other potentially meaningful stimuli (Federmeier 2007; Kutas and Federmeier 2000, 2011; Molinaro et al. 2010). N400 amplitude is sensitive to a variety of stimulus and context manipulations, including word frequency, repetition, sentence and discourse congruity, lexical association, concreteness and semantic richness, semantic processing load and semantic priming (Federmeier and Laszlo 2009; Kutas and Federmeier 2011). N400 amplitude is considered an index of the difficulty of accessing and retrieving stored conceptual knowledge associated with a word (Barber and Kutas 2007; Duncan et al. 2009; Kutas and Federmeier 2000; Voss and Federmeier 2011). Given its sensitivity to various semantic manipulations such as word repetition, it has been suggested that the N400 may serve as an effective dependent variable for studying semantic memory and recognition memory (Kutas and Federmeier 2011).

Besides N400, ERP studies identified the visual N1 and P2 components as relevant to early lexical processing, semantic access, and word recognition (Breznitz 2002; Segalowitz and Zheng 2009; Sereno and Rayner 2003; Sereno et al. 2003). The N1 is a negative-going polarity peaking between 100 and 200 ms over posterior scalp locations, that is considered an index of selective attention and thought to reflect visual discrimination processes (e.g., Vogel and Luck 2000). The N1 attention effect seems to be more prominent when subjects are required to discriminate between stimuli than when they must merely detect the presence of a stimulus (Vogel and Luck 2000). Selective attention mechanisms regulate behavioral responses through enhancement of the processing of relevant information while suppressing the processing of irrelevant information. Selectivity prevents reflexive reactions to stimuli in the environment and allows for behavioral flexibility (Boxem et al. 2005; Vogel and Luck 2000; Wijers et al. 1996). The P2 is a positive-going component occurring approximately 200-300 ms post-stimulus onset. It originates in the inferior occipital (extrastriate) cortex and has been identified in many different cognitive tasks, including stimulus classification/discrimination, response inhibition, selective attention, and short-term memory (Barry et al. 2003; Crowley and Colrain 2004; Key et al. 2005). It was also reported that P2 can be modulated by the valence of stimuli in the context of affective tasks, being most pronounced in response to unpleasant/negative visual stimuli (Carretié et al. 2001; Delplanque et al. 2004). In lexical tasks, both N1 and P2 have shown sensitivity to word frequency and predictability and to word emotional valence (Carreiras et al. 2005; Herbert et al. 2006; Kuchinke et al. 2015; Lee et al. 2012; Scott et al. 2009; Sereno et al. 2003).

Our research hypotheses were as follows: At the behavioral level, drawing on prior studies suggesting that visual motor speed processing is adversely affected during pregnancy (Crawley et al. 2008; Henry and Sherwin 2012; Raz 2014; Fiterman and Raz 2019), we expected slower reaction times (RTs) among pregnant women compared with controls. Based on the findings of Henry and Rendell (2007) and Sharp et al. (1993), we did not anticipate a main effect of group on accuracy (percent of errors). At the electrophysiological level, considering the nature of the task (visual recognition with emotional and neutral words as stimuli) and informed by the literature (e.g., Carreiras et al. 2005; Kutas and Federmeier 2011; Lee et al. 2012; Sereno et al. 2003), we focused on the N1, P2, and N400 ERP components at anterior and posterior-parietal scalp locations. We expected that pregnant women, compared to non-pregnant women, will show differences in the mean amplitudes of these selected components. Additionally, we anticipated that ERPs will interact with the type of word stimuli; specifically, we hypothesized that pregnant women will exhibit more pronounced neural responses to emotional words, particularly those with negative emotional content (Pearson et al. 2009).

## Methods

### Participants

Participants included 22 pregnant women in the third trimester of pregnancy and 25 non-pregnant controls matched to the pregnant group for age, number of children, and educational level (see Table 1 for sample characteristics). All participants shared the same ethnicity and native language. The sample predominantly consisted of college academic staff (~ 45%) and college administrative staff (~ 16%), along with undergraduate and graduate students (~ 28%), ensuring equal proportions between the two groups. Participants were recruited through advertisements displayed across the campus and via the college's internal email system. The remaining small percentage in both groups was achieved through individuals who encountered the advertisements and existing participants who helped recruit their eligible acquaintances who met the study's inclusion criteria. Participants had no history of neurological or psychiatric conditions. All of them had normal or corrected-to-normal visual acuity. Inclusion criteria for the pregnancy group were: ≥ 18 years of age, gestational age ≥ 26 weeks, singleton pregnancy, having normal current pregnancy and without a history of adverse pregnancy-related conditions or terminations. In both groups, women were excluded from the study if they had children younger than 1 year of age. Written informed consent was obtained from all participants. All subjects participated voluntarily. The study was approved by the Max Stern Yezreel Valley College review board.

**Table 1:** Sample characteristics

	Pregnant (*n* = 22)			Non- pregnant (*n* = 25)			*p*
	Mean	SEM	Range	Mean	SEM	Range	
Age (years)	33.36	0.97	23–42	31.88	1.22	22–44	0.21
Education (years)	17.59	0.62	12–21	17.20	0.47	13–21	0.47
Number of children	0.82	0.19	0–2	1	0.23	0–3	0.55
Gestational age (weeks)	32.95	0.82	26–37	-	-	-	-

### Measures

#### Emotional word recognition task

**Fig. 1:**
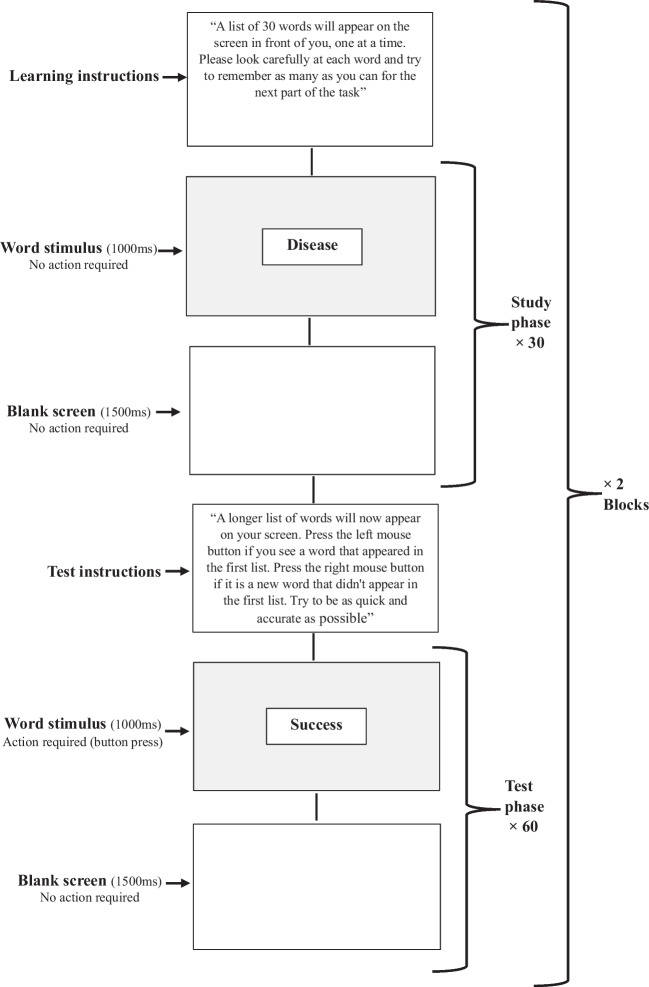
Schematic description of the emotional word recognition task

We used a classic Old/New word recognition task (Johnson et al. 1985) consisting of a test phase in which subjects are asked to judge whether visual stimuli were previously presented in an initial learning phase ('old') or not ('new'). Due to the length of this task, it was divided into two blocks of 90 items, with each block consisting of 30 words in the learning phase, followed by a recognition test consisting of 60 words (30 taken from the original study list and 30 new words). In each block, participants were first presented with 30 words, one word at a time, at the center of a computer monitor. A third of the words were emotionally neutral (e.g., the Hebrew version for: "door”, “car”, “shoe”), a third were emotionally negative (e.g., the Hebrew version for: "rape", “pain”, “murder”) and a third were emotionally positive (e.g., the Hebrew version for: "happiness", “hug”, “success”).^1^ Words ranged between three and six letters. Each condition (negative/positive/neutral) consisted of equal number of short (3 letters), medium (4–5 letters), and long (6 letters) words. All trials consisted of a 1000 ms stimulus display followed by a blank screen for an inter-trial interval of 1500 ms. During this phase, participants were instructed to study the words without any active response. Subsequently, participants performed a recognition test in which they were presented with 60 words (half were taken from the previously studied list i.e., 'old', and half were not previously presented i.e., 'new') and were required to judge whether each word had appeared previously in the learning phase or not. Again, a third were neutral, a third positive and a third negative. Responses were made by pressing the left or right button of the computer mouse. Participants were allowed a short rest period between the two blocks and were informed that there is no connection between the two lists of words in each block. The order of the blocks was counterbalanced across participants to mitigate order effects at the block level. During the learning stage, words were presented in a random order. In the recognition test phase, to minimize potential expectancy effects, words from the various conditions within each block (i.e., old-negative, old-positive, old-neutral, new-negative, new-positive, and new-neutral) were also presented in random order, ensuring unpredictability in presentation. See Fig. 1 for a schematic description of the task.

### Procedure

Upon arrival at the lab, participants completed a demographic and personal details questionnaire and a computerized emotional word recognition task with EEG-ERP recording. During the EEG-ERP session, the subjects were seated in an armchair, 80 cm away from a 19" computer screen. During the learning phase of the task, participants were instructed to carefully watch the list of word stimuli presented at the center of the screen and try to remember them for the next stage of the task. In the recognition test phase, they were instructed to indicate as quickly as possible, but without compromising accuracy, whether or not each presented word had been previously shown in the initial list. Responses were made by pressing the left button (for an 'old' word) or the right button (for a 'new' word) on the computer mouse. RTs and error rates were recorded.

### EEG recording; data acquisition and target-evoked ERP components

**Fig. 2:**
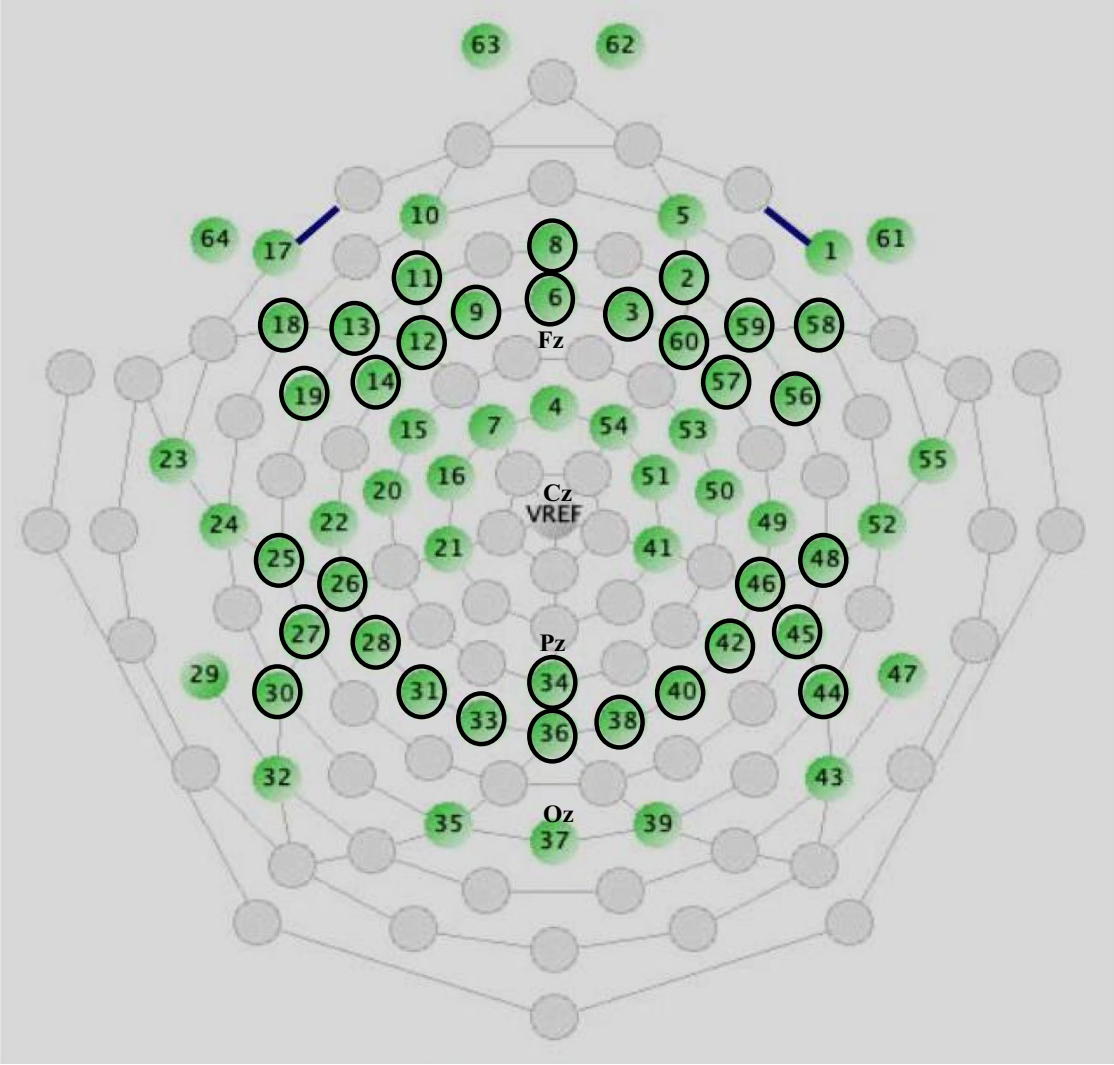
Schematic representation of the EGI 64 HydroCel Geodesic Sensor Net and the electrodes chosen for analyses

EEG was recorded continuously as participants engaged in an emotional word recognition task using a 64-channel HydroCel Geodesic Sensor Net, Net Amps 300 amplifier, and Net Station, Version 4.2, software (Electrical Geodesics Inc, Eugene, OR) at 250 Hz with 0.1 Hz high-pass and 100 Hz low-pass filtering. Electrode impedances were maintained below 50 kΩ. During acquisition, all channels were referenced to the vertex electrode. All stimulus presentations and behavioral response collections were controlled by a PC computer running E-prime 2.0 software (Psychology Software Tools Inc., PA). Data preprocessing was performed offline following acquisition using NetStation, (Waveform Tools Package), Version 4.2, software (Electrical Geodesics Inc, Eugene, OR). Continuous EEG was filtered with a 1–30 Hz band-pass filter and segmented by condition into 900 ms stimulus-locked epochs (i.e., time-locked to stimulus onset) going from 100 ms pre-stimulus to 800 ms post-stimulus. Segments contaminated with vertical eye movements (eye blinks; ± 140 µV) and horizontal eye movement (± 55 µV) artifacts, as automatically identified by the built-in NetStation artifact detection tool, were eliminated. Following the manufacturer's recommendation and as is customary in other studies that used the same system (e.g., Alderman et al. 2015; Bai et al. 2015; Bayet et al. 2021; Chung et al. 2018; Fournier et al. 2021; Key and Jones 2019; Killebrew et al. 2018; Vanderwert et al. 2015; Wang and Han 2014), recording segment was marked as 'bad' if it contained ten or more bad channels (15% of the total number of electrodes; bad channel: ± 200 μV for the entire segment). Individual bad channels were replaced on a segment-by-segment basis using the built-in NetStation bad channel replacement tool. Participants were excluded from further analysis if they had fewer than 85% good trials per experimental condition, which result in a minimum of 34 trials per condition. The average percentage of artifact-free trials did not significantly vary between groups (pregnant 86.5%, non-pregnant 88.8%) or conditions. Averaged ERP data was baseline corrected (100 ms pre-stimulus onset) and re-referenced into an average reference frame. N1 (120-180 ms post-stimulus), P2 (180-320 ms post-stimulus) and N400 (320-500 ms post-stimulus) components were chosen for analyses based on inspection of the grand average ERPs of both the pregnant and non-pregnant groups, and the above-mentioned a-priori hypotheses. Considering scalp topography distributions and in alignment with existing research on ERPs during recognition tasks —particularly those focusing on the frontal N400 (FN400) and parietal N400 old/new effects—we analyzed pre-selected ERPs at both the frontal (average of channels 2, 3, 6, 8, 9, 11, 12, 13, 14, 18, 19, 56, 57, 58, 59, 60) and parietal (average of channels 25, 26, 27, 28, 30, 31, 33, 34, 36, 38, 40, 42, 44, 45, 46, 48) scalp regions (Curran et al. 2006; Griffin et al. 2013; Nyhus and Curran 2009; Rugg and Curran 2007; Šoškić et al. 2021; Voss and Paller 2009).^2^ For the electrode array, see Fig. 2.

### Data analysis

Data analysis was performed using SPSS 25.0 (IBM Corp 2017).

#### Behavioral data analysis

To examine whether pregnant women, compared to control group, react differently to 'old' and 'new' negative, positive and neutral words, group differences and interaction effects in error rates and RTs were analyzed using a 2 × 2 × 3 repeated measures (mixed-design) ANOVA with Group (Pregnant/Non-pregnant) as the between-subject factor, and Condition (Old/New) and Emotional Content (Negative/Positive/Neutral) as the within-subject factors. Independent sample t-tests were used for post-hoc comparisons.

#### ERP analysis

To assess the relationship between pregnancy and brain activity (N1, P2, N400) in the learning stage, we performed a 2 × 3 (Group × Emotional content) mixed-model ANOVAs. In the recognition test phase, preliminary 2 × 2 × 3 analyses (Group × Condition × Emotional content) yielded only marginally significant results, likely due to the relatively small number of trials, which did not provide sufficient statistical power (with a maximum of 20 trials per condition). Consequently, for further ERP data analysis, 'old' and 'new' stimuli were combined into a single general variable of Emotional Content (Negative/Positive/Neutral), and negative, positive, and neutral stimuli were grouped into a single general variable of Condition (Old/New). Separate 2 × 3 (Group × Emotional Content) and 2 × 2 (Group × Condition) mixed-model ANOVAs were then conducted to analyze the mean amplitudes of the selected N1, P2, and N400 ERP components.

In both the behavioral and ERP analyses, significance levels were adjusted using the Huynh–Feldt correction when needed (the degrees of freedom indicated in the text are always those before the Huynh–Feldt correction, but the p-values are always those after the correction). Follow-up independent sample t-tests were used to break down interaction effects. The Bonferroni correction was applied and only results that remained significant after the correction are reported. A total of 42 subjects were included in behavioral and ERP analyses (Pregnant = 22, non-pregnant = 20). Five participants from the non-pregnant group were excluded due to excessive artifacts in the ERP data (fewer than 85% good trials per experimental condition). Throughout the “Results” section, numeric and graphical results are presented as mean $\pm$ SEM (the standard error of mean).

## Results

### Behavioral results

**Fig. 3:**
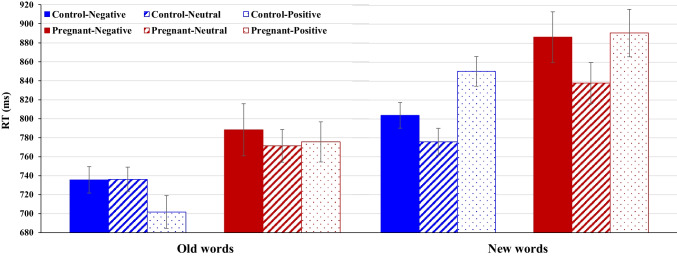
Response times (RT; in milliseconds) for pregnant and control groups to negative, neutral, and positive words in the Old and New conditions

A 2 × 2 × 3 repeated measures ANOVA revealed a significant interaction between Group, Condition, and Emotional Content [F_(2,80)_ = 4.27, *p* = 0.017, *ηp*^*2*^ = 0.10]. Within this framework, there was a significant Condition × Emotional Content interaction [F_(2,80)_ = 21.29, *p* < 0.0001, *ηp*^*2*^ = 0.35], manifesting as notable differences in response times (RTs) to the assessed emotions in the 'new' words condition (*p* < 0.0001), but not in the 'old' words (*p* = 0.13). To further elucidate this interaction within the context of the three-way dynamic, separate 2 × 2 ANOVAs for each group confirmed its presence in both pregnant [F_(2,42)_ = 13.94, *p* = 0.007, *ηp*^*2*^ = 0.26] and non-pregnant groups [F_(2,38)_ = 22.88, *p* < 0.0001, *ηp*^*2*^ = 0.55]. Additionally, the main effect of Condition was robust across groups [F_(1,80)_ = 86.39, *p* < 0.0001, *ηp*^*2*^ = 0.68], showing longer RTs to ‘new’ versus ‘old’ words in both pregnant [F_(1,42)_ = 40.29, *p* < 0.0001, *ηp*^*2*^ = 0.66] and non-pregnant groups [F_(1,38)_ = 50.23, *p* < 0.0001, *ηp*^*2*^ = 0.73]. Importantly, a significant main effect of Group was observed [F_(1,40)_ = 6.02, *p* = 0.019, *ηp*^*2*^ = 0.13], with pregnant women exhibiting significantly slower RTs (825.13 ms ± 20.08) compared to controls (767.26 ms ± 14.58). This effect remained consistent and did not significantly interact with Condition or Emotional Content, underscoring distinct group-level differences that manifest irrespective of experimental conditions or emotional contexts (Fig. 3). Given the complexities of the three-way interaction, while it suggests further nuances that merit exploration, conducting such an exhaustive analysis exceeds the current study's scope and primary aims. Therefore, it was not pursued further.

No significant 2 × 2 × 3 repeated measures ANOVA was found for the measure of Accuracy (percent of errors). Pregnant and non-pregnant women did not differ with respect to error rates, and there were no Group × Condition, nor Group × Emotional content interaction effects. Taken together, test performance results indicate that pregnant women were slower to react but as accurate as non-pregnant controls.

### ERP results

Table 2 summarizes the significant between-group and interaction ERPs effects in the learning stage, recognition test-emotional content (old/new combined), and recognition test-condition (negative/positive/neutral combined), at anterior and posterior channels.

**Table 2:** Summary of significant between-group and interaction ERPs effects in the learning stage, recognition test-emotional content (old/new combined), and recognition test-condition (negative/positive/neutral combined), at anterior and posterior channels

	Learning stage				Recognition test- emotional content (Old/New combined)				Recognition test- condition (Negative/Positive/Neutral combined			
	Between-group effect		Group × Emotion interaction effect		Between-group effect		Group × Emotion interaction effect		Between-group effect		Group × Emotion interaction effect	
ERP component	Anterior	Posterior	Anterior	Posterior	Anterior	Posterior	Anterior	Posterior	Anterior	Posterior	Anterior	Posterior
N1	0.036	0.025	N.S	N.S	0.023	0.017	N.S	0.049	0.027	0.007	0.049	0.042
P2	0.022	0.027	N.S	N.S	0.019	0.043	N.S	N.S	0.005	0.016	N.S	N.S
N400	0.007	0.016	N.S	N.S	0.023	0.012	0.039	N.S	0.021	0.017	N.S	N.S

Numbers represent the *p* value

#### Learning stage

**Fig. 4:**
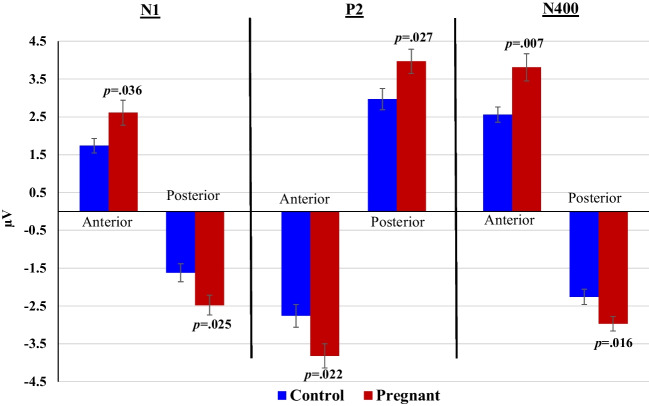
Mean amplitudes of N1, P2 and N400 at anterior and posterior channels by pregnant and non-pregnant groups during the learning stage of the task (no active response is required in this stage)

Analysis of ERPs during the passive learning stage of the task revealed that pregnant women had significantly more pronounced N1 [anterior: F_(1,40)_ = 4.69, *p* = 0.036, *ηp*^*2*^ = 0.11; posterior: F_(1,40)_ = 5.45, *p* = 0.025, *ηp*^*2*^ = 0.12], P2 [anterior: F_(1,40)_ = 5.68, *p* = 0.022, *ηp*^*2*^ = 0.12; posterior: F_(1,40)_ = 5.28, *p* = 0.027, *ηp*^*2*^ = 0.12], and N400 [anterior: F_(1,40)_ = 8.18, *p* = 0.007, *ηp*^*2*^ = 0.18; posterior: F_(1,40)_ = 6.30, *p* = 0.016, *ηp*^*2*^ = 0.14] compared with non-pregnant controls (Fig. 4). Group did not interact with Emotional content.

#### ERP results- recognition test phase- emotional content (Old/New combined)

**Fig. 5:**
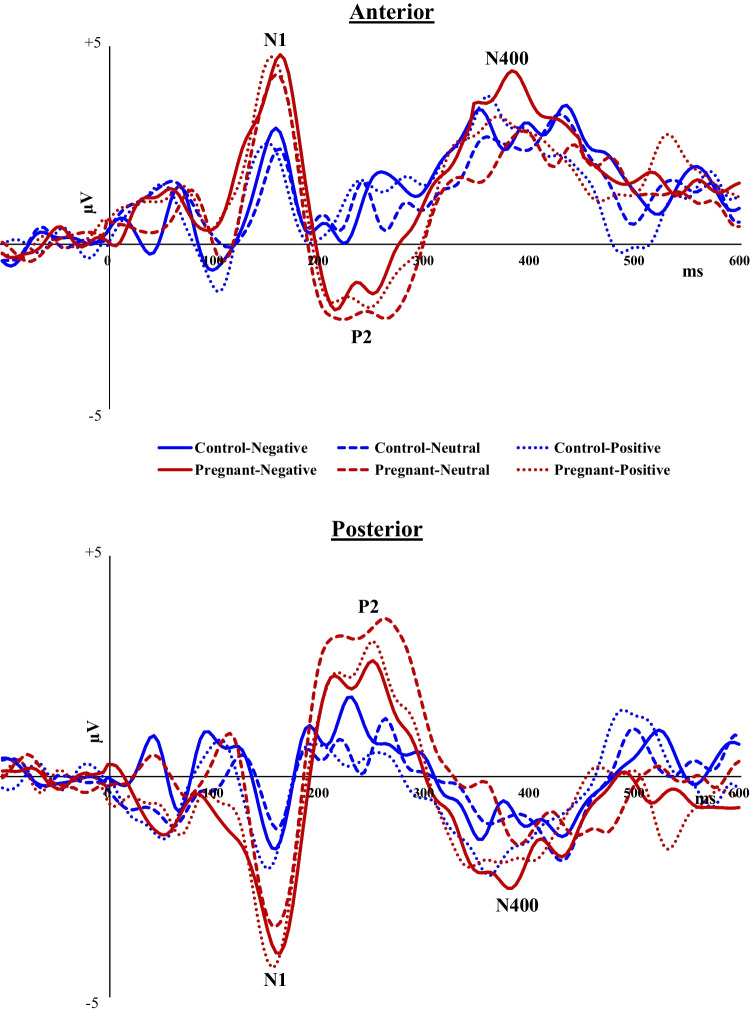
Grand averaged ERPs to negative, neutral and positive words by pregnant and non-pregnant groups at anterior and posterior channels

Figure 5 depicts the grand averaged ERPs to negative, neutral and positive words by pregnant and non-pregnant groups at anterior and posterior channels.

##### N1 (120-180 ms post-stimulus)

**Fig. 6:**
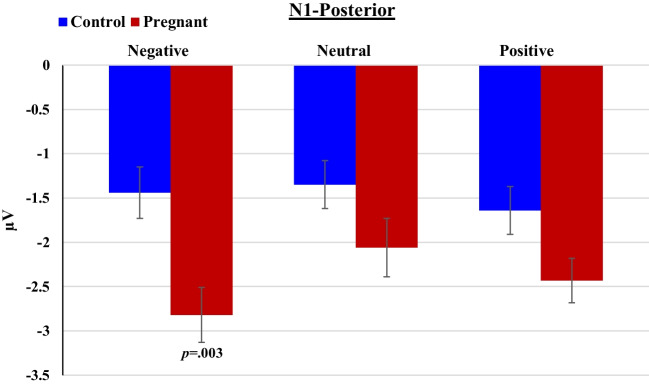
Mean amplitudes of the N1 component to negative, neutral and positive words by pregnant and non-pregnant groups at posterior channels

Analysis for the anterior cluster revealed a significant Group difference in N1 amplitude [F_(1,40)_ = 5.56, *p* = 0.023, *ηp*^*2*^ = 0.12], such that pregnant women had more pronounced N1 (2.58 µV ± 0.33) compared to non-pregnant women (1.62 µV ± 0.28). No interaction was found between Group and Emotional content. At posterior channels, analysis revealed a main effect of Emotional content [F_(2,80)_ = 4.71, *p* = 0.012, *ηp*^*2*^ = 0.11]- N1 to negative and positive words was greater than to neutral words; a main effect of Group [F_(1,40)_ = 6.25, *p* = 0.017, *ηp*^*2*^ = 0.14]- pregnant women had more pronounced N1 than controls; and, a Group × Emotional content interaction [F_(2,80)_ = 3.14, *p* = 0.049, *ηp*^*2*^ = 0.07]. Follow-up comparisons showed that pregnant women had significantly more pronounced N1 to emotional negative words than non-pregnant women [t(40) =  − 3.18, *p* = 0.003, Cohen’s d = 0.99], while no such significant group difference was found for neutral (*p* = 0.12) and positive (*p* = 0.044) words (Fig. 6).

##### P2 (180-320 ms post-stimulus)

Analysis for both the anterior and posterior locations revealed a main effect of Group [F_(1,40)_ = 5.97, *p* = 0.019, *ηp*^*2*^ = 0.13; F_(1,40)_ = 4.35, *p* = 0.043, *ηp*^*2*^ = 0.10, respectively] such that pregnant women had greater P2 amplitude (anterior: -2.35 µV ± 0.28; posterior: 2.72 µV ± 0.35) compared with non-pregnant controls (anterior: -1.56 µV ± 0.19; posterior: 1.91 µV ± 0.22). No interaction effects were found between Group and Emotional content.

##### N400 (320-500 ms post-stimulus)

**Fig. 7:**
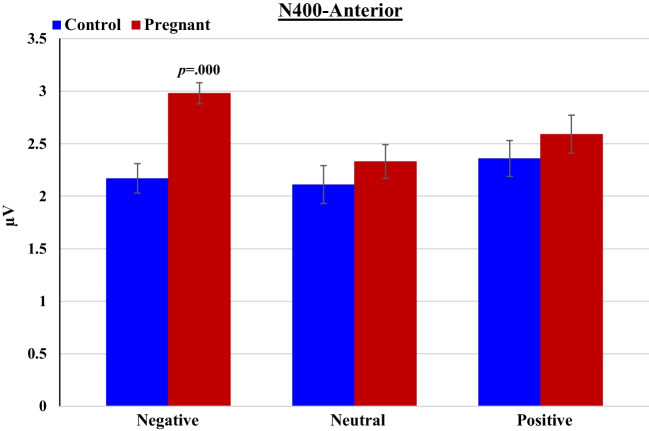
Mean amplitudes of the N400 component to negative, neutral and positive words by pregnant and non-pregnant groups at anterior channels

Analysis for the anterior channels revealed a main effect of Emotional content [F_(2,80)_ = 4.26, *p* = 0.024, *ηp*^*2*^ = 0.10]- N400 was more pronounced to negative than to neutral words; a main effect of Group [F_(1,40)_ = 5.60, *p* = 0.023, *ηp*^*2*^ = 0.12]- pregnant women had more pronounced N400 than non-pregnant women; and, a significant Group × Emotional content interaction [F_(2,80)_ = 3.67, *p* = 0.039, *ηp*^*2*^ = 0.08]- i.e., Group difference in N400 amplitude was evident only for negative words [t(_40_) = 4..44, *p* = 0.0001; Cohen’s d = 1.38] and not for neutral (*p* = 0.37) or positive (*p* = 0.36) words (Fig. 7). At posterior channels, there was a main effect of Emotional content [F_(2,80)_ = 4.10, *p* = 0.02, *ηp*^*2*^ = 0.09]- N400 to negative and neutral words was greater than to neutral words. There was also a main effect of Group [F_(1,40)_ = 6.89, *p* = 0.012, *ηp*^*2*^ = 0.15]: Pregnant women had greater (more negative) N400 (-2.10 µV ± 0.16) than non-pregnant women (-1.63 µV ± 0.15). The interaction between Group and Emotional content did not reach statistical significance.

#### ERP results- recognition test phase- condition (Negative/Positive/Neutral combined)

**Fig. 8:**
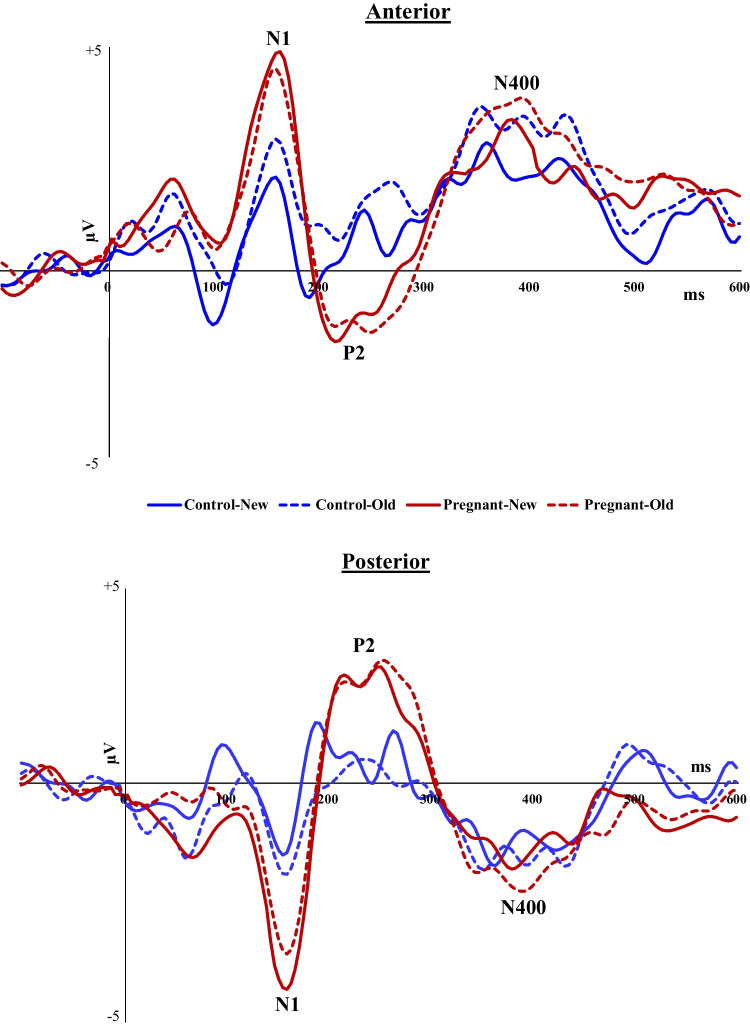
Grand averaged ERPs to Old and New words by pregnant and non-pregnant groups at anterior and posterior channels

Figure 8 depicts the grand averaged ERPs to Old and New words by pregnant and non-pregnant groups at anterior and posterior channels.

##### N1 (120-180 ms post-stimulus)

**Fig. 9:**
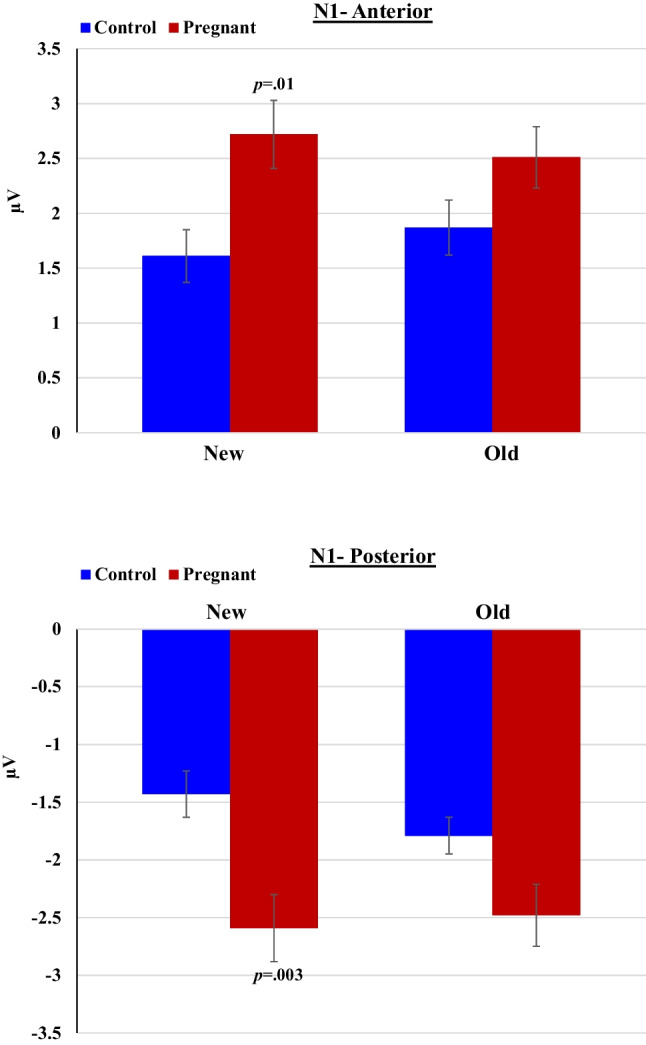
Mean amplitudes of the N1 component to Old and New words by pregnant and non-pregnant groups at anterior and posterior channels

Analysis for anterior channels showed a significant Group difference in N1 amplitude [F(_1,40_) = 5.26,*p* = 0.027, *ηp*^*2*^ = 0.12], such that pregnant women had more pronounced N1 than controls; as well as a Group × Condition interaction effect [F(_1,40_) = 4.11,*p* = 0.049, *ηp*^*2*^ = 0.09]: the difference in N1 between pregnant and controls was significant only for New words [t(40) = 2.70, *p* = 0.01, Cohen’s d = 0.84] but not for Old words (*p* = 0.10). Similar results were found for posterior channels: Pregnant women had greater N1 than controls [F(_1,40_) = 8.01,*p* = 0.007, *ηp*^*2*^ = 0.17], and there was a significant interaction between Group and Condition [F(_1,40_) = 4.43,*p* = 0.042, *ηp*^*2*^ = 0.10]. The difference in N1 between pregnant and non-pregnant women was evident especially in response to New words [t(40) = -3.21, *p* = 0.003, Cohen’s d = 1.00] compared to Old words (*p* = 0.04) (Fig. 9).

##### P2 (180-320 ms post-stimulus)

Analysis of both the anterior [F(_1,40_) = 8.24,*p* = 0.005, *ηp*^*2*^ = 0.18] and posterior F(_1,40_) = 6.27,*p* = 0.016, *ηp*^*2*^ = 0.14] locations showed a significant effect of Group, such that pregnant women had more pronounced P2 (anterior: -2.23 µV ± 0.27; posterior: 2.54 µV ± 0.26) compared with controls (anterior: -1.33 µV ± 0.15; posterior: 1.74 µV ± 0.19). There were no interactions between Group and Condition.

##### N400 (320-500 ms post-stimulus)

Analysis of both the anterior [F(_1,40_) = 5.76,*p* = 0.021, *ηp*^*2*^ = 0.13] and posterior F(_1,40_) = 6.25,*p* = 0.017, *ηp*^*2*^ = 0.14] locations showed a significant effect of Group, such that pregnant women had more pronounced N400 (anterior: 2.43 µV ± 0.13; posterior: -1.89 µV ± 0.14) compared with non-pregnant women (anterior: 2.00 µV ± 0.15; posterior: -1.46 µV ± 0.12). No Group × Condition interactions were found.

## Discussion

In this study, we investigated behavioral and brain function measures to assess cognitive-affective processing in pregnant women in their third trimester, compared to non-pregnant control women. We examined the neurological correlates of pregnancy by recording ERPs during a visual emotional word recognition task, which assesses declarative memory and early processing of verbal emotional content. Behaviorally, our hypothesis that pregnant women would perform with comparable accuracy but slower reaction times than non-pregnant controls was supported. These results align with previous findings indicating slower reaction times in late pregnancy (Crawley et al. 2008; Henry and Sherwin 2012; Raz 2014; Fiterman and Raz 2019) and are consistent with studies showing no accuracy differences in recognition tasks between pregnant women and controls (Henry and Rendell 2007; Sharp et al. 1993). Contrary to common subjective perceptions among pregnant women, our results suggest that recognition ability, in terms of accuracy, is not impaired during late pregnancy. However, maintaining accuracy levels comparable to non-pregnant women comes at the cost of significantly slower reaction times.

At the electrophysiological level, we hypothesized that pregnant women would show differences from non-pregnant controls in the mean amplitudes of several task-related ERP components, focusing on N1, P2, and N400. During the learning/study phase of the task, where participants were instructed to observe and memorize target words, pregnant women exhibited larger N1, P2, and N400 amplitudes at both anterior and posterior sites. This may indicate that the pregnant group required additional processing compared to the non-pregnant group. In the recognition test phase, which required an active discriminating response, we combined 'old' and 'new' stimuli into one general variable of Emotional content (negative/neutral/positive). Here, our results again showed significant group differences in all pre-selected components at anterior and posterior channels. Pregnant women displayed more pronounced mean amplitudes of N1, P2, and N400 than non-pregnant women. Moreover, for N1 and N400, significant interactions were observed between pregnancy status and the emotional content of words. N1 (posterior) and N400 (anterior) were significantly greater in pregnant women for emotionally negative words, whereas no such difference was evident for emotionally positive and neutral words (similar interactions for anterior N1 and posterior N400 were also noted but did not reach the required statistical significance after Bonferroni correction). This study's observation of a pregnancy-related selective neural response to negative content is consistent with findings by Pearson et al. (2009). They reported that women in late pregnancy showed increased accuracy in recognizing emotional expressions that signal threat or harm, such as fearful, angry, and disgusted faces. Similarly, Roos et al. (2012) found that pregnant women exhibited significantly heightened attention to fearful faces compared to non-pregnant women, suggesting an enhanced sensitivity to danger cues during pregnancy. This heightened awareness is thought to be an adaptive response, emphasizing the importance of parental precaution and responding to potential hazards during pregnancy. Our study corroborates these findings, demonstrating a similar heightened response in pregnant women to emotional negative words (lexical stimuli), rather than to visual images.

When negative, positive, and neutral stimuli were consolidated into a single variable of Condition (old/new), our findings once again demonstrated that pregnant women exhibited more pronounced mean amplitudes of N1, P2, and N400 compared to non-pregnant women, at both anterior and posterior locations. Additionally, an interaction was observed between pregnancy status and the old/new condition for N1. Specifically, the N1 amplitude in pregnant women was significantly greater than that in controls, but only in response to 'new' words; no such difference was observed for 'old' words. Taken together, our study reveals a distinct pattern of increased neural reactivity in late pregnancy, particularly marked by heightened responses to both emotionally negative and novel word stimuli during the recognition task.

N1, P2 and N400 components are highly relevant to processing of visual verbal information (Breznitz 2002). The visual N1 component is thought to reflect early perception, selective attention, and the operation of a discrimination process within the focus of attention (Luck 2012; Vogel and Luck 2000). It has been suggested that greater N1 amplitude may reflect generally greater sensory sensitivity or increased arousal (Burkhart and Thomas 1993). N1 amplitude is also affected by perceptual load, thus larger N1 is typically seen in tasks that require greater allocation of perceptual processing resources (Vogel and Luck 2000). Early ERP effects around 100 ms post stimuli, during a word recognition task, are proposed to index early attentional resource allocation to rapidly process potentially meaningful information (Citron 2012). The P2 ERP component is thought to index the recruitment of early attentional resources that forms a basis for subsequent cognitive processing. It has been identified in tasks involving short term memory and stimulus classification (Barry et al. 2003; Crowley and Colrain 2004; Key et al. 2005). Fiterman and Raz (2019) found that pregnant women, compared to controls, had greater P2 amplitude in response to target stimuli during a stop signal task. N1 and P2 amplitudes are also sensitive to word stimuli emotional content; larger N1 and P2 are evident in response to emotional relative to neutral words (Herbert et al. 2006; Kanske and Kotz 2007; Kanske et al. 2011; Kissler et al. 2007; Scott et al. 2009). The N400 component reflects a neural response to words in all modalities. It is thought to index the difficulty of retrieving stored conceptual knowledge associated with a word and is modulated in amplitude when prior exposure turns semantic processing to easier (Kutas and Federmeier 2000; Kutas and Federmeier 2011). There is an ongoing debate in the literature, in several respects, regarding the interpretation of the N400 effect in the context of semantic processing during recognition tasks. For instance, some researchers draw a distinction between two hypothesized processes that contribute to performance in tests of recognition memory- familiarity and recollection, and suggest that these processes are reflected in two distinct ERP effects: frontally distributed FN400 reflecting familiarity-based recognition, and parietal N400 reflecting semantic processing (e.g., Bridger et al. 2012; Eichenbaum et al. 2007; Mecklinger 2000; Paller et al. 2007; Rugg and Curran 2007; Stróżak et al. 2016). Other researchers doubted this discrimination and claimed that frontal N400 and parietal N400 are actually electrophysiologically and functionally identical (e.g., MacKenzie and Donaldson 2007; Voss and Paller 2009; Voss and Federmeier 2011). Either way, it has been shown that N400 amplitude is modulated by a wide variety of stimulus and context factors (e.g., priming manipulations, semantic context, word frequency, lexical class in sentences; as well as the specific method of EEG recording, referencing and ERP analysis). Going into a more thorough review of the N400 literature is beyond the scope of this paper and exceeds the immediate goals of the present study (for review see: Bornkessel-Schlesewsky and Schlesewsky 2019; Cheyette and Plaut 2017; Kutas and Federmeier 2011; Lau et al. 2008).

Augmentation of N1, P2 and N400 may reflect the recruitment of additional brain resources for perceptual processing of attended emotional stimuli. Such augmentation in ERP responses may suggest that women in late pregnancy had to recruit additional brain processing resources in order to successfully perform the task, and may partly explain their slower response times. The greater N1, P2 and N400 amplitudes found in pregnant women relative to controls may indicate that they are generally more alert, and specifically more sensitive and reactive to novel and emotional negative signals in their environment.

The present research has some limitations that deserve to be addressed. First, our pregnancy sample included both multigravid and primigravid women. Although the groups were closely matched in terms of motherhood status (with 50% of those in the pregnancy group and 52% in the control group being mothers) and the number of children—thereby underpinning the credibility of these findings—there is still a possibility that experiences of motherhood might have slightly affected the outcome patterns. Consequently, future studies may attempt to further control for reproductive history and parenthood status, for example, by specifically targeting primigravid and nulliparous women or by drawing comparisons between primigravid and multigravid participants. Second, we investigated women during late pregnancy. It would be of interest to explore the development of ERP differences from early to late pregnancy as well as from late pregnancy to postpartum. Third, the sample size in this study, while typical for ERP research (Clayson et al. (2019)), was relatively small. The majority of participants, being undergraduate/graduate students and college administrative and academic staff, possessed a relatively high level of education. This characteristic could potentially influence their attitudes, motivation, performance, and compliance during the experimental sessions. To enhance the generalizability of our results and expand our understanding, future studies should aim for larger sample sizes and strive for replication with samples that are more representative of diverse educational backgrounds, socioeconomic statuses, and ethnicities. Finally, the limited number of trials in each condition of the task seemed to restrict our ability to achieve statistically significant results in robust ERP analyses. Consequently, we opted to conduct separate analyses for the combined variables of Emotional Content and Condition. Future studies should aim to strike a more effective balance between task duration/cognitive load and the number of trials needed for valid statistical ERP analysis.

In conclusion, our results shed new light on memory function and neural activity during late human pregnancy. Contrary to popular belief, pregnant women did not differ from non-pregnant women in their memory task performance (i.e., accuracy levels achieved). They did, however, differ from non-pregnant women in RTs and in their neural response patterns. To our knowledge, this study is the first to examine both behavioral and neural measures of recognition memory and related early processing of verbal emotional content, during late pregnancy, using event related potentials. The current results may suggest that at early stages of neural processing, women at late pregnancy are generally more hypervigilant to novel environmental stimuli and are particularly more sensitive to stimuli with negative content. Heightened precautionary behavior, reflected, among other things, in an increased sensitivity towards environmental novel stimuli and towards signals of threat and harm may be advantageous in optimizing fetal growth and development and preparing women for the unique demands of motherhood (Anderson and Rutherford 2012; Hahn-Holbrook et al. 2011; Pearson et al. 2009).

## Data Availability

Due to privacy and ethical concerns the data generated during this study can be provided by the corresponding author upon reasonable request and with necessary approvals.

## Footnotes

1 In a small preliminary study, our research team selected a list of words categorized as negative, neutral, or positive. Participants, who were not involved in the main study, rated each word on a scale from 1 (negative) to 7 (positive) for emotionality (valence), and from 1 (low) to 7 (high) for arousal. We calculated average rankings for each word, excluding those that did not conform to their expected categories: negative words scoring above 2, neutral words falling outside the 3–5 range, and positive words below 6. Words lacking sufficient inter-judge reliability were also disqualified. Using inferential statistics, including ANOVA and subsequent pairwise comparisons, we confirmed significant differences between the categories. This ensured that words were accurately categorized by emotional tone and that comparable levels of arousal were maintained between the negative and positive words.

2 Polarity conventions at the field of ERP studies are highly inconsistent. ERP waveforms can be plotted with upward deflections indicating positive or negative potentials at the active electrode relative to the reference electrode. Both conventions are used in the literature and no consensus exists as to which is preferable. When comparing waveforms to those in the literature, it is essential to consider differences in recording system and reference method (Duncan et al. 2009; Picton et al. 2000). In this study, all channels were referenced to the vertex sensor during acquisition and then re-referenced into an average reference frame. This resulted in negative going posterior N1 and N400 and positive going posterior P2 with a corresponding frontal/anterior polarity reversal mirroring posterior effects.
